# Medicine Men of the World

**Published:** 1888-03-10

**Authors:** 


### Medicine Men of the World.

By the Man in the Crowd.

(*Continued from page* 308.)

VIII.

A curious Japanese tradition makes a Chinese physician the source of the first elements of civilisation among them. It was in the days of Sikono, a tyrannical Emperor of China, who, after drinking of all the pleasures of life to satiety, began, after the manner of roués and rakes in general, to be troubled occasionally with very bad fits of the blues. Life was very short, his magnificence and power were very great; what a pity to leave it all! Could not some of the philosophers about him find some panacea against death? His chief physician thought there could be no doubt that such a thing could be found. The ingredients for it were he was convinced to be found in the islands far away under the rising sun. Let only the resplendent Son of Heaven give his learned physician the opportunity, and he would go forth and gather these ingredients; only, he must take with him three hundred youths and as many maidens, all healthy in body and vigorous in mind, for the materials for such a panacea as the Emperor required were subtle and delicate herbs, and could be gathered only by chaste hands.

The youthful band of six hundred, with the great physician at their head, set out for the islands beneath the rising sun, equipped with everything they could possibly demand, at the Emperor's expense. And so they sailed away and established themselves in Japan. The physician had begun to feel that under such a tyrant his head was anything but secure, and he had devised this simple ruse as a convenient method of getting out of his power. The Japanese, says Mr. Eden, still show the part of the coast where the Chinese physician landed, and point out the stones of the temple erected of old in his honour as a reward for his having introduced the first elements of civilisation and of the arts and sciences.

This immigration from China has been sometimes represented as the founding of the Japanese nation. It certainly was not so, however; but it may, no doubt, be regarded as the founding of "the faculty" in Japan. Hence, no doubt, it is that medicine and medicine men in Japan have, up till the past few years at least, been just about what we have already described them to be in China. The medical profession is sometimes hereditary, but it is open to all who choose to adopt it, and therefore brings no great wealth or credit as a rule. There are three grades of physicians: those who attend upon the Court exclusively, the army doctors, and the general practitioners, who find their patients where they can. The old medical science of Japan was very largely made up of sorcery and humbug, and to a very large extent was practised by blind men. These belonged to a common class of the profession, whose sole art consisted in performing a kind of shampooing, something like that practised at Turkish baths, a process by which these primitive professors would undertake to cure any of the many ills to which flesh is heir. They, however, were very generally recognised as mere quacks; and another section of the profession, apparently on about the same professional level, were wont to adopt as their panacea the practice of acupuncture. When there was a pain these doctors would stretch the skin and thrust in a fine sharp-pointed needle. Another common remedy for all sorts of maladies, from a fatty tumour to an attack of cholera, was cauterisation. "Cauterisation," says Mr. Evans, "is performed with little cones called 'moxas,' formed of dried wormwood leaves, and prepared in such a manner as to consume slowly. One or more of these is applied to the diseased part and set alight." If a bad attack of the gout in a man's big toe could not be expelled in this way, it is really very difficult to say what severe measures could be resorted to with any probability of success. A certain degree of medical knowledge is very generally diffused among the Japanese. "They have," says the authority just quoted, "anatomical models in cardboard, painted a flesh colour, upon which the spinal column and other salient points of the human form are distinctly marked, with characters or numbers attached to them corresponding with similar numbers in a book which contains a full and detailed account of every disease that especial part is addicted to, and instructions how to prick or cauterise it, and how often. Nearly every Japanese family possesses this species of medical manual, and such is the reputation of the remedies therein contained, that many people make use of them at fixed periods, and as preventives." Nevertheless, doctors abound in Japan, and there seems to be hardly any species of imposture and quackery that has not been practised by them from time immemorial.

But in this respect, as in so many others, the Japanese have of late years been making marvellous changes. How far they may have carried them by this time, and as to how far a spirit of the times has made its influence felt in the interior of the country, it is not easy to say. Fifty years ago the love of novelty and the spirit of inquiry, so singularly wanting in the Chinese, but so wonderfully characteristic of the Japanese now that they have once thrown open their country to the influence of foreigners, had evidently begun to stir the medical profession in Japan. A traveller at that time told us how he had sat before a lattice, which screened the Emperor from his sight, and had been questioned in all sorts of ways as to the practice of European doctors in this, that, and the other matter. What were the external and internal distempers which Europeans found most difficult to cure? How did they proceed in cases of cancerous tumours and imposthumes of the inner parts? Did not the European doctors search after medicine to make people immortal? Had they made any progress in their search? Which was the most important medicine Europeans had yet discovered? Could the traveller make up the medicine himself? No! Well, where could it be procured? And so on. That cross-examination by the Emperor seems to have been typical of what was stirring in the more intelligent section of Japanese society at that time, and it has borne very important fruits. A writer in the *Atlantic Monthly* some few years back gave a very interesting account of the opening of the first private hospital in Japan under the direction of Dr. Matsumoto, a native, eminent for his scientific attainments, and who had abandoned the cherished system of his ancestors and struck boldly out into the paths of European pathological science. A slight but interesting account of the hospital follows, and the writer closes his paper by a literal translation of a short drama with which the inaugural day was appropriately brought to a close, and which, as indicating the tendency of things in the medical world of Japan, is certainly very suggestive and interesting.

Neurosis comes upon the stage richly dressed as a monarch, with a crown and sceptre, and strides about the stage in great agitation. He is followed by Paralysis and Pyrosis. They complain that their power is declining. Doctors are arising of such skill as to combat them all, and they concert plans for the dissemination of disease with such violence that none can be cured. While they talk there are cries from without from Science, who exclaims, "I surrender! I surrender!" Like most of his brethren, he says, he was born and reared in blindness, and his life has been passed in fighting blindly against disease, but now disease is becoming too strong, and he wishes to join their party. He will, however, pretend to be still their enemy, but he will so manage things that disease shall thrive. Then comes Truth, who also desires to join the society of disease. Science and Truth are, in fact, only counter-plotting against Neurosis, Pyrosis, and Paralysis. They drug and master the Genii of Disease.

"You have deceived us," cries Neurosis. "We believed you were our confederate. Or is it all a jest?"

"It is no jest," says Truth. "I have used a stratagem to destroy you."

"And I am Science," says the other. "Truly, in former times I was nothing but a blind shampooer, but that is all changed now."

